# Reports on the Hospitals of the United Kingdom

**Published:** 1910-01-08

**Authors:** Henry Burdett


### REPORTS
ON THE
### Hospitals of the United Kingdom.

By SIR HENRY BURDETT, K.C.B., K.C.V.O.

### XVIII.—THE ROYAL INFIRMARY, LIVERPOOL.

This institution commenced as a Public Infirmary in 1745, its object being the treatment of all poor, diseased, or hurt persons, not only in Liverpool, but from all parts of the nation and Ireland. It is interesting to note that the original site granted by the Corporation on lease for 999 years was, in fact, that upon which St. George's Hall now stands. Seventy-one years afterwards the Corporation desired this site for its own use, and as the original hospital had become inadequate a new site in Brownlow Street was granted by the Corporation. Liverpool men have deservedly held a high reputation for their business qualities, and the managers of the Royal Infirmary in 1820 obtained an annuity from the Corporation of £210 in addition to the new site. The Derby family have always been good friends of the Royal Infirmary. The stone of the new infirmary of 1821 was laid by Lord Stanley, and that of the present hospital building was laid by the Earl of Derby in 1887, and his successor laid the foundation stone of the new out-patient department in July 1909.

### The Growth in the Cost of Hospital Buildings.

It may be interesting to trace the growth in the cost of hospital buildings and the corresponding decrease in the building value of the British sovereign as illustrated by the history of this institution. The original building opened in 1749 cost £2,618; the new building opened in 1824 cost £20,426; and the present building, opened in 1890, cost £181,000. The present hospital contains 300 beds, of which 270 are constantly occupied. Of course the population of Liverpool has increased enormously during the 70 years' interval, but, making due allowance for the increased accommodation, it must be apparent to every thoughtful mind how enormously great is the increase in the present cost of providing permanent buildings for modern hospital purposes. Another fact which it may be well to drive home in this connection is that the more efficient the modern hospital becomes in every department, the more certain is it that extended buildings become essential every decade at least. Liverpool must therefore not be surprised that the able board of managers of the Royal Infirmary have found it necessary that an improved out-patient department shall be built without delay. This, it is estimated, will cost some £30,000. With that splendid munificence which has been exhibited from time to time by the merchant princes of Liverpool, the Bickersteth family have given £10,000 in memory of the late E. R. Bickersteth, some members of whose family we have had the pleasure of working with in the past. We were delighted to find one of them at work in the operation theatre when we visited that unit of the hospital. In the course of our tour of inspection we have met with other examples of the continuous association with a hospital of members of a family during succeeding generations, to the great advantage and benefit of each institution. We found one eminent surgeon, for instance, rendering yeoman service to a Poor Law Infirmary at no doubt great personal cost to himself, because he is attached to the place by associations, and so this institution has received not only important personal services, but valuable aid which otherwise it might have been impossible for it to secure.

It is an interesting, and we are inclined to think a unique fact that the Corporation of Liverpool, despite all that has been said against ground landlords of late, has invariably extended generous, material, and financial aid to the Royal Infirmary whenever large building operations have had to be faced. Opposed as we are on principle to the handing over of our great voluntary hospitals to the municipalities and so permitting them to become rate-supported institutions, we believe that the Corporation of Liverpool has shown a way to every municipality in the country whereby each Corporation can generously support the building operations of the local voluntary hospital, and so save the ratepayers' pockets, whilst it ensures its efficient management by upholding the voluntary principle of hospital support. We have seldom read a terser, more businesslike, or convincing appeal than that issued in support of the new out-patient department, with which we shall deal later. We hope that all the money now required will be speedily subscribed.

### The Evolution of our Hospitals.

Another very interesting feature of the Royal Infirmary is the fact that, like some of the older Metropolitan and a few noteworthy provincial and Scotch hospitals, it had connected with it in 1830-34 a lunatic asylum and a Lock hospital. The City and County Councils have taken over the charge of lunatics, and the Poor Law authorities the Lock cases; but the public spirit exhibited by the former managers of the Royal Infirmary was abundantly rewarded in 1881, when the land on which the lunatic asylum stood was acquired by the railway, and so formed the nucleus, and a good nucleus too, of the building fund for the new infirmary.

Again, Liverpool has always been to the fore in matters of nursing. There Agnes Jones of blessed memory did a memorable work, and Mr. Rathbone made a reputation second only to the great Lord Shaftesbury by his splendid efforts in connection with nurses, their training and development. The Nurses' Home in Ashton Street is justly claimed by the Liverpool authorities as a pioneer institution in the movement which has given to all classes of the community the blessing of an adequate system of trained nurses of the best type. It will be seen, then, that if the spirit which has dominated the citizens of Liverpool for upwards of a century and a half, in regard to hospital and nursing matters, is still extant, the present Royal Infirmary, its buildings, its administration, and its work must be judged by the highest standard.

### The Royal Infirmary of To-day.

Impressed by this feeling, we devoted several hours to a close inspection and examination of every detail of the work and of many parts of the buildings. We purposely postponed our visit until we had had an opportunity of inspecting the more recently erected hospitals, as well as some of the older hospitals in this district of the country, so that we might be in the best position to judge where the Royal Infirmary, Liverpool, is entitled to stand to-day. There is one grave defect, which is relatively a small one, and is, we believe, due to an oversight on the part of the architect or of those who were responsible for furnishing some of the requirements of the new institution when the plans were made. That defect is the inadequate and unworthy accommodation provided for the wardmaids—in cubicles, the majority of which have not direct communication with the outside air, and no window of their own. We mention this because wardmaids are not able to take care of themselves, and it seems to us a great pity that this relatively small matter should not be set right immediately, for the reason that an otherwise nearly perfect plan is marred. In all other respects the plan exhibits a knowledge and thoroughness in work which has, unfortunately, too seldom been exhibited by the modern architect entrusted with a great hospital building. Our experience is that architects fail, and very often in limited and other competitions the assessors fail too, to give due justice to the plans submitted in competition, because they almost necessarily lack the technical knowledge which enables them to realise what the requirements of a modern hospital are, and that it is essential that every new hospital shall be something better than a mere aggregate of units of points in existing hospitals. If two assessors were always appointed as they ought to be, one an architect and the other a man of full knowledge and experience of hospital requirements and development, the waste of immense sums of public money which now unfortunately takes place might be avoided to a great extent. It is heart-breaking to inspect hospital after hospital and to find it is not up to date however recent its buildings may be. A general hospital is not a town or village, but a great sick house for the treatment of acute diseases and accidents. What may be suitable accommodation and arrangements for the purpose of a hospital for infectious diseases, for administrative, economical, and hygienic reasons may certainly not be suitable or even desirable for a great voluntary hospital. Alfred Waterhouse, R.A., who designed the present Royal Infirmary, was a man who believed in the doctrine of thorough. This principle is written upon every part of the Royal Infirmary plan, and although the present buildings are 20 years old, they contain practically every facility and accommodation afforded by the most recent hospital buildings in this district of England. With its advantages for administrative reasons, the Waterhouse plan, by its thoroughness and understanding of the details, places this hospital to-day deservedly first amongst the voluntary hospital buildings in this part of the country. Waterhouse was a pioneer, and, possibly, for want of practical working experience, the diameter of his circular wards is a little greater than it need be. But the more this plan is studied and the more experience of the working of this hospital the managers have, the stronger becomes the evidence that Waterhouse's plan for a hospital of this size is remarkably good and up to date.

### The Spirit of the Hospital.

We have said that this hospital must be judged by the highest standard of efficiency, and too few people realise what that represents in these days, when trained nursing and the altar to cleanliness which asepsis has erected in every efficient hospital have made such institutions a marvel of hygienic perfection in these matters. It is necessary to insist that millions may be spent on hospital buildings, that the money for maintenance may overflow in abundance, that every appliance and fitting, appurtenance and requisite may be of the best, and yet a hospital may fail from the inefficiency in its administration. The spirit of efficiency in every department, a knowledgable superintendent and matron, the best of surgeons and physicians, and the most carefully trained and capable of nurses all working loyally together, impelled by the same spirit to do their best and to see that everything down to the smallest detail is the best which they collectively and individually can attain, make the perfect modern hospital. Expressed in the daily life of a large hospital, this spirit produces, even in great city hospitals, an almost perfect atmosphere, because ventilation and absolute cleanliness are everywhere maintained. It means a thorough mastery of the best appliances and methods whereby the pressure of the work can be eased as much as possible, and the most satisfactory results secured in the shortest possible time. It means a place for everything and everything in its place; the most perfect orderliness and neatness, and the absence in daily use of any substance like turpentine or spirits of salt in the cleansing of baths and lavatories, because powdered bath-brick sprinkled on a scouring cloth rinsed in hot water and blue mottled soap produces better results at a nominal cost. It means constant, unending, alert inspection of every part of the hospital, and the consideration, nay the anticipation, of the just requirements and needs of every department and each individual, so that the whole establishment runs its daily course as smoothly and naturally as if it were a nicely adjusted machine. Judged by this standard, we have great pleasure in expressing our opinion that the Royal Infirmary, Liverpool, may fairly take its place as, on the whole, one of the best administered, most healthy, attractive and efficiently administered hospitals not only in the North of England but in the country. All honour to the men and women who have produced this result. They deserve recognition at the hands of the citizens of Liverpool. What more practical recognition can be given them than the immediate supply of the relatively small sum of money which the managers need to finish and equip the new out-patients' department, and to make the maintenance fund sufficient to fulfil its objects?

THE NEW OUT-PATIENT DEPARTMENT.

We have had an opportunity of going over the plans and arrangements of the new out-patient department. These plans, like the appeal already referred to, do credit to the business instincts and technical knowledge of those who are responsible for them. In one sense the available site is none too large, in another we are glad it is so restricted, because it has rendered it essential that the best brains available should be put into the plans and arrangement of the new department, with results which we venture to think will give general satisfaction. The arrangements throughout are well thought out. The plan of restricting the size of the central hall by providing a waiting-room in connection with the unit of each department is a good one, and has the merit of novelty. If it succeeds, as we believe it will succeed, this out-patient department may mean a new departure in hospital construction. At any rate, the minds which have produced the absence of overcrowding by successfully handling upwards of 20,000 out-patients, representing 80,000 attendances each year, within the present limited accommodation, will, we are confident, ensure the smooth working of the new out-patient department, and make it yield results which may be memorable in the history of hospital construction. We had the advantage and pleasure of having with us during our inspection Miss E. M. Jones and Mr. Francis H. Moore. We fear the length of time occupied may have well night exhausted their patience, but in thanking them for the great courtesy and assistance they rendered, we venture to hope that a perusal of this report may convince them that it could not have been otherwise written. Our object was to form a mature judgment of the Royal Infirmary, Liverpool, as it exists to-day, and we claim that its present splendid efficiency merits everything we have here set forth.

Finally, we would like to congratulate Mr. Moore upon the artistic cover of the 160th report for 1908, and on the report itself. It is attractive in appearance and the type is legible, a point which is sometimes forgotten. An annual report must be read to be effective, and to be read fruitfully it must be printed in a type clearly legible to advanced sight.

### XIX.—THE GLOUCESTERSHIRE ROYAL INFIRMARY AND EYE INSTITUTION.

THIS hospital contains 140 beds, of which about 90 are constantly occupied on an average. It is nineteen years since we last inspected this institution. At that time (in THE HOSPITAL, October 1890) we recorded that we found an excellent spirit of friendly rivalry in efficiency in all departments of this hospital. At the present time this spirit prevails with, if possible, greater fervour, and the whole establishment is remarkable by reason of the bright, smart, orderly appearance which is everywhere apparent. Much of the furniture is made from oak, presented to the hospital by King George III., and most of the floors are now teak. The furniture and floors alike are kept in as perfect order as some old and treasured dining-table in a careful household. We are naturally in favour of glass tops for ward-tables and lockers, in surgical wards especially. In Gloucester the authorities cling with affectionate tenacity to the polished oak tables of King George III., and these tables certainly present an attractive appearance, the pattern of the grain in the wood presenting a most attractive picture. One sister has an artistic eye, and her ward was made especially attractive by having tables of various heights down the centre of the ward on which ferns and flowers were arranged. This hospital, which was instituted in 1755, gives another proof of the admitted fact that nowadays, under the aseptic system, old buildings when ably administered can be kept as hygienic and efficient for the treatment of disease as the newest of wards.

There are, of course, spots on the sun, and at Gloucester the General Committee should no longer tolerate the unsightly and imperfect slop-sinks and other fittings and apparatus in the sculleries and lavatories. Indeed, it is not just to the members of a hospital staff as efficient as that at Gloucester to permit these out-of-date appliances longer to spoil the excellent impression created in the mind of a visitor by the present high efficiency of the management. There is a further need for reconstruction in the surgical wards on both sides in the operation block, where the central fireplaces are unsightly, inadequate, and out of date. The operation theatre, too, excellent as it is, in many respects lacks adequate means whereby the temperature can be raised to the necessary extent. We believe that a sum of £2,000 to £3,000 would defray the cost of this reconstruction and new equipment. Seeing that the invested property exceeds £75,000, and that the annual expenditure is under £8,000 per annum, the General Committee is justified in proceeding with the reconstruction and improvements we recommend, which are urgently called for, without further delay. The institution presents throughout, with the exceptions in question, the appearance of being thoroughly looked after by the board, who evidently take a pride in making it efficient. We have good reason, therefore, to express confidence that Colonel Curtis-Hayward and his committee will no longer tolerate the continuance of the unsightly and serious, though relatively small, defects to which we have ventured to call their attention.

A MARK OF ROYAL APPROVAL.

In June last King Edward, on the occasion of his visit to Gloucestershire, conferred a mark of his favour and recognition on this institution. Henceforward this hospital will be known as the Royal Infirmary.

**The Cheltenham General Hospital and Dispensary** will be reported on next week.

